# Supplementary material for: Non-invasive fibrosis algorithms are clinically useful for excluding cirrhosis in prisoners living with hepatitis C
Source: PLoS One. 2020 Nov 18;15(11):e0242101. doi: 10.1371/journal.pone.0242101 (PMC7673506; doi:10.1371/journal.pone.0242101)
Supplement: S1 Table — (DOCX) [file pone.0242101.s001.docx]

# Supplemental table 1:

Supplemental table 1: Accuracy of FIB4 in predicting significant fibrosis as compared to transient elastography

|  |  | Liver stiffness measurement | |  |  |  |  |
| --- | --- | --- | --- | --- | --- | --- | --- |
|  | All prisoners  (n=1007)  n (%) | < 9.5kPa  (n=791)  n (%) | ≥ 9.5kPa  (n=216)  n (%) | Sensitivity  % | Specificity  % | PPV  % | NPV  % |
| For prediction of advanced fibrosis |  |  |  |  |  |  |  |
| FIB4 |  |  |  |  |  |  |  |
| ≤1.45 | 713 (71) | 635 (80) | 78 (36) | 64 | 80 | 47 | 89 |
| >1.45 | 294 (29) | 156 (20) | 138 (64) |  |  |  |  |
| ≤ 3.25 | 960 (95) | 785 (99) | 175 (81) | 18 | 99 | 87 | 82 |
| > 3.25 | 47 (5) | 6 (1) | 41 (19) |  |  |  |  |

Supplemental table 1: The sensitivity, specificity, NPV and PPV of FIB-4 for advanced fibrosis (LSM ≥ 9.5kPa) compared with TE. Legend, PPV, positive predictive value, NPV, negative predictive value, TE, transient elastography, LSM, liver stiffness measurement.
