# Supplementary material for: Non-invasive fibrosis algorithms are clinically useful for excluding cirrhosis in prisoners living with hepatitis C
Source: PLoS One. 2020 Nov 18;15(11):e0242101. doi: 10.1371/journal.pone.0242101 (PMC7673506; doi:10.1371/journal.pone.0242101)
Supplement: S2 Table — (DOCX) [file pone.0242101.s002.docx]

# Supplemental table 2:

Supplemental table 2: Performance of novel fibrosis assessment pathway applied to a validation cohort.

|  |  | Liver stiffness measurement | |  |  |  |  |
| --- | --- | --- | --- | --- | --- | --- | --- |
|  | All prisoners  (n=189)  n (%) | < 12.5kPa  (n=169)  n (%) | ≥ 12.5kPa  (n=20)  n (%) | Sensitivity  % | Specificity  % | PPV  % | NPV  % |
| For prediction of cirrhosis |  |  |  |  |  |  |  |
| Age + APRI |  |  |  |  |  |  |  |
| >35 years and APRI ≥1.0 | 33 (17) | 18 (11) | 15 (75) | 75 | 89 | 45 | 97 |
| ≤35 or >35 and APRI <1.0 | 156 (83) | 151 (89) | 5 (25) |  |  |  |  |

Supplemental table two: Sensitivity, specificity, PPV and NPV of fibrosis assessment pathway incorporating age and APRI thresholds. Legend, PPV, positive predictive value, NPV, negative predictive value
